# Important Decision in the Celluloid Case

**Published:** 1876-02

**Authors:** 


### SELECTED ARTICLES.

### ARTICLE VI.

OFFICE OF THE CELLULOID MANUFACTURING Co,
Newark, N. J., December 28th, 1875.

S. S. WHITE, Esq., Philadelphia.

DEAR SIR.—The inclosed letter of Mr. Hamilton, addressed to me, explaining the case of Goodyear Dental Vulcanite Company and Josiah Bacon against Flagg, will give you the situation better than if I undertook the explanation myself. You are probably aware that the movement of Josiah Bacon in Baltimore last week, in which an attempt was again made to introduce the subject of Celluloid in connection with the vulcanite rubber dental plates, and which they were obliged to withdraw as soon as we appeared by counsel, is another evidence of their attempts, by all underhanded means, to steal a march upon us, instead of meeting the issue square upon its merits.

Yours truly,
MARSHALL LEFFERTS, *President.*

---

New York, December 23d, 1875.

MARSHALL LEFFERTS, Esq., *President of the Celluloid Manufacturing Company.*

DEAR SIR—The Goodyear Dental Vulcanite Company and Josiah Bacon have this day voluntarily withdrawn the suit against Dr. Eben M. Flagg, which they brought, you will remember, for the purpose of enjoining Dr. Flagg from the use of Celluloid in making dental plates, and have consented to pay Dr. Flagg's costs rather than pursue the case any further.

In view of the long-continued threats by Mr. Bacon and his Company to prosecute and punish dentists daring to use Celluloid for dental plates, it is proper that you should be informed of the circumstances under which this suit was begun and ended. It was begun under the most favorable circumstances for the Goodyear Company and Mr. Bacon. They had several years in which to collect their evidence and to prepare for trial. They chose their own court and their own place of trial, their own defendant and their own time. They chose to begin the suit in midsummer, when lawyers are absent from the city on their vacation. They could not have a better case against any one than they had against Dr. Flagg, as it was admitted beyond question and in the clearest manner that he had for over a year used Celluloid in making dental plates, and had infringed the Cummings patent, if the use of Celluloid constituted any infringement.

If the complainants sincerely desired a test case this surely was a favorable one for them. The Celluloid Company were glad of this opportunity to finally put an end to the threats of the Goodyear Company and Mr. Bacon.

The complainants at the commencement of the suit made a motion before Judge Blatchford, in New York, for a preliminary injunction, using on that motion the evidence they had so long been accumulating,—using also the affidavits of Josiah Bacon; of G. H. P. Flagg, a dentist of Boston; of Dubois B. Parmlee, a chemist; of Henry B. Renwick, an expert; and of Henry I. Fisk.

They also referred to the printed record in the Smith case and to the previous decisions in their favor, and they presented to the court, as exhibits for examination, metal dental plates, vulcanite dental plates, and Celluloid dental plates. In short, it seems that the complainants used on that motion everything of fact or of law that could, in the opinion of their experienced counsel, aid them in any way.

The motion was argued by Messrs. E. N. Dickerson and B. F. Lee, for the complainants, and by Judge William D.

Shipman, Clarence A. Seward, and E. Luther Hamilton, for Dr. Flagg. Elaborate printed briefs were submitted to Judge Blatchford, and while he had the matter under consideration, Dr. Flagg, who was anxious to bring the case on for a speedy trial, applied to the Court and obtained an order to compel the complainants to put in their proof by a certain fixed day.

The time fixed by that order expired this afternoon.

On December 7th, Judge Blatchford rendered his decision denying their motion for a preliminary injunction, and filed the following opinion in the case:

U. S. Circuit Court, Southern District of N. Y.

The Goodyear Dental Vulcanite Co., and others *vs.* Eben M. Flagg.

Blatchford, J.

"I do not find that any decision has been made in regard to the plaintiff's patent which gives to it such a construction as necessarily includes the process and substance used by the defendant. In the Gardner case the defendant did not compound India-rubber with sulphur, but he compounded India rubber with iodine, and he employed heat to harden the rubber. (*Goodyear Dental Vulcanite Company* vs. *Gardner*, 4 *Fisher's Patent Cases*, 224, 231.)

"In the Smith case the view of the Court was that the material to be used under the plaintiff's patent in carrying out the invention patented was to be India-rubber, 'and the compounds commonly employed therewith reduced to a soft plastic condition, capable of vulcanization and subsequently vulcanized.' (*Goodyear Dental Vulcanite Company* vs. *Smith*, 5 *Official Gazette of Patent Office*, 585.)

"It appears from the description of the process used by the defendant in this suit, that he does not use India-rubber or any substance capable of vulcanization. That the substance he uses is one which is rendered plastic by heat, and is not hardened by heat. That heat is used in the process to soften the substance and render it plastic and not to harden it, and that the substance after being moulded is hardened by being cooled. It is not sufficiently clear that this process is embraced in the claim of the plaintiff's patent to warrant the granting of an injunction until one is awarded as the result of a decree for the plaintiffs on final hearing."

Instead of proceeding with the case and putting in their proofs as the Court has ordered them to do, the complainants this afternoon discontinued and dismissed the suit, and consented to pay Dr. Flagg's costs rather than to go on. The complainants have thus chosen to back out of a suit instituted by themselves and brought, as they pretended, to test the question between vulcanite and Celluloid. Judge Blatchford's decision on their motion was the shadow of the coming event, which no doubt induced them to withdraw the suit.

(Signed) Yours truly,

E. Luther Hamilton, *Solicitor for Dr. Flagg.*

—*Dental Cosmos.*

